# Parent Anxiety and Response to a Telehealth Intervention for Hypoglycemia Fear: Secondary Analysis of a Randomized Trial

**DOI:** 10.2196/95475

**Published:** 2026-11-03

**Authors:** Susana R Patton, Alexandra Monzon, Nicole Kahhan, Jacob Redel, Kimberly Driscoll

**Affiliations:** 1 Center for Healthcare Delivery Science, Nemours Children's Clinic, 807 Children's Way, Jacksonville, FL, 32207, United States, 1 904-697-3595, 1 904-697-3425; 2 Center for Healthcare Delivery Science, Nemours Children's Clinic, Orlando, FL, United States; 3 Division of Psychology, Nemours Children's Clinic, Jacksonville, FL, United States; 4 Division of Endocrinology, Children's Mercy Hospital, Kansas City, MO, United States; 5 Center for Diabetes Technology, University of Virginia, Charlottesville, VA, United States

**Keywords:** pediatrics, type 1 diabetes, hypoglycemia fear, parenting, anxiety

## Abstract

**Background:**

Parents of young children with type 1 diabetes (T1D) frequently report fear of hypoglycemia (FH), which can impact their diabetes management. REDCHiP (Reducing Emotional Distress for Childhood Hypoglycemia in Parents) is a telehealth-delivered cognitive behavioral intervention shown to reduce FH, but less is known about which parents benefit most.

**Objective:**

This secondary analysis examined whether pretreatment parent anxiety moderated REDCHiP’s effects and explored responder patterns by intervention arm and pretreatment anxiety.

**Methods:**

This was a secondary analysis of a large, randomized clinical trial comparing REDCHiP to an attention control condition (ATTN). We assessed outcomes before treatment (time 1) and 3 months after treatment (time 3). Our primary outcome was parental FH at time 3, adjusted for time 1 FH. The original trial used validated measures of parent FH and general anxiety. We evaluated parent anxiety at time 1 as both a categorical variable (clinically elevated or not) and a continuous variable (centered). We defined responders as parents achieving a reduction of ≥0.5 SDs in FH from time 1 to time 3. An analysis of covariance tested the treatment arm by anxiety interaction, with sensitivity analyses modeling anxiety continuously.

**Results:**

There was no significant treatment arm by anxiety interaction when modeling parent anxiety as either a categorical or continuous variable (*P*>.05). However, exploratory analyses found that the adjusted mean time 3 FH was numerically lower among REDCHiP parents than ATTN parents with clinically elevated anxiety. Among parents with elevated anxiety, 78.6% (22/28) of REDCHiP parents met our responder criterion compared with 47.4% (9/19) of ATTN parents. Among parents with elevated anxiety, the adjusted odds of meeting the responder criterion were higher for REDCHiP than ATTN parents (odds ratio 6.35, 95% CI 1.47‐29.00).

**Conclusions:**

While time 1 parent anxiety did not significantly moderate treatment response, exploratory analyses suggested a potentially meaningful pattern among parents with clinically elevated anxiety. As telehealth-delivered behavioral interventions expand in pediatric type 1 diabetes care, secondary analyses of participant characteristics may help guide targeted implementation and enhance treatment benefit.

## Introduction

Parents of young children with type 1 diabetes (T1D) commonly report fear of hypoglycemia (FH) [1,2], which may adversely influence their treatment decisions and consequently young children’s glycemic control [2]. To address this concern, we designed REDCHiP (Reducing Emotional Distress for Childhood Hypoglycemia in Parents), a telehealth-based intervention to reduce parent FH [3]. Telehealth-delivered treatments may be more scalable for families managing T1D because they can reduce barriers associated with traditional in-person treatments, including transportation demands, time and scheduling constraints, and childcare responsibilities [4-7]. However, as the availability of telehealth interventions continues to expand [4,7], it is essential to identify which families are most likely to benefit so that we can implement programs efficiently and target the families with the greatest need.

Parent anxiety is an established correlate of FH [8-10], and higher anxiety may amplify the constant vigilance, worry, and avoidance behavior that characterizes FH. Because REDCHiP teaches parents cognitive and behavioral strategies for managing FH worry and reducing their reliance on potentially harmful hypoglycemia avoidance behaviors [3], it is possible that parents’ pretreatment anxiety symptoms could influence their engagement with or responsiveness to treatment.

We recently completed a large 2-arm randomized clinical trial comparing REDCHiP to a study-specific attention control condition (ATTN) [11]. In the main trial, parents in both treatment arms experienced a significant reduction in FH [12]. Here, we present secondary analyses exploring pretreatment parent characteristics that may have been associated with parent treatment response. Specifically, we examined whether parents’ pretreatment anxiety moderated reductions in parent FH. We also explored response patterns among participants by treatment group and their pretreatment anxiety level.

## Methods

### Ethical Considerations

We conducted the primary multisite clinical trial under a single institutional review board (IRB) agreement, with Children’s Mercy Hospital-Kansas City as the IRB of record. Before enrolling parents, we obtained IRB approval on all study procedures, including the use of electronic documentation of parent informed consent and permission. The primary trial was registered at ClinicalTrials.gov (NCT03914547).

### Study Design

We previously described the primary trial’s inclusion and exclusion criteria, study procedures, and trial outcomes [11,12]. We randomized families following enrollment. We delivered the REDCHiP and ATTN treatments entirely via video telehealth following guidelines for best practices in digital behavioral health [13]. Both treatment arms had 10 sessions delivered nearly weekly over 12 weeks. We assessed parent outcomes at before treatment (time 1), immediately after treatment (time 2) and 3 months after treatment (time 3). To be consistent with the primary analyses from the REDCHiP trial, these secondary analyses also focused on outcomes at time 3 to examine maintenance of treatment effects.

### Measures

#### Overview

All participating parents completed the same questionnaires online [11,12]. At time 1, we used a trial-specific questionnaire to capture family demographics, including parent and child age, sex, and race and ethnicity. We also asked parents to describe their relationship to the child, marital status, and education level, as well as provide information on their child’s T1D, including date of diagnosis, treatment regimen, and history of severe hypoglycemia.

#### Hypoglycemia Fear

Parents completed the validated Hypoglycemia Fear Survey–Parents of Young Children (HFS-PYC) [2]. This questionnaire measures parents’ FH based on the frequency of hypoglycemia-related worries and avoidance behaviors. Items used a 5-point Likert scale (1=never to 5=very often), with higher scores indicating greater FH. To simplify score interpretation, the primary trial transformed HFS-PYC scores to fit a 0 to 100 scale [12]; we applied the same rescoring method to the current analyses. To calculate a responder variable, we defined treatment response as parents who achieved a reduction of ≥0.5 SD from time 1 to time 3 using the time 1 HFS-PYC SD from the full sample [14]. We used this distribution-based approach because a minimal clinically important difference has not yet been established for the HFS-PYC [15].

#### General Anxiety

Parents completed the PROMIS-A (Patient-Reported Outcomes Measurement Information System Anxiety Short Form). This is a validated 7-item questionnaire assessing physical and cognitive symptoms of general anxiety over the past 7 days. Items used a 5-point Likert scale reflecting the frequency or intensity of symptoms. We selected parent anxiety as a candidate moderator based on theory and empirical findings linking elevated anxiety to greater FH [8-10]. We examined parent anxiety at time 1 as both a continuous variable, to capture the full range of symptom severity, and as a categorical variable based on normative guidelines to identify parents with or without clinically elevated anxiety (PROMIS-A T-scores ≥60) [16].

### Statistical Analysis

Our primary model was an analysis of covariance examining time 3 parent FH. The model included treatment arm, time 1 anxiety (elevated versus not elevated), and the treatment arm by anxiety interaction as fixed factors and time 1 parent FH as a covariate. We also conducted a sensitivity analysis that modeled parent PROMIS-A T-scores as a continuous, centered variable, and retested the treatment arm by anxiety interaction. Finally, we explored responder rates by time 1 anxiety (elevated versus not elevated) by treatment arm. Among parents with elevated anxiety, we used logistic regression to explore whether responder rates differed by treatment arm after adjusting for time 1 parent FH. We conducted all analyses using SPSS (version 27; IBM Corp).

## Results

Of the 183 families randomized, 152 (83%) completed time 3, including 77 and 75 families randomized to REDCHiP and ATTN, respectively (Figure 1). Thirty-one families were lost to follow-up during the primary trial. There were no significant differences in time 1 demographics, child clinical characteristics, or parent-reported FH and general anxiety among those who completed the trial versus those who withdrew (Multimedia Appendix 1).

**Figure 1.**
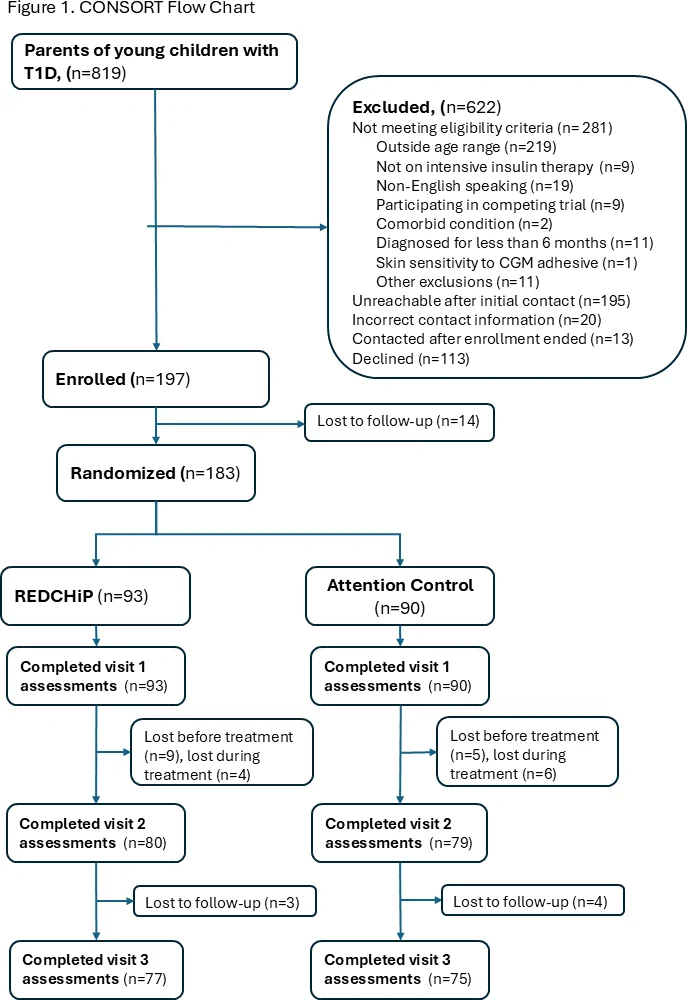
CONSORT (Consolidated Standards of Reporting Trials) flowchart. REDCHiP: Reducing Emotional Distress for Childhood Hypoglycemia in Parents; T1D: type 1 diabetes.

Table 1 summarizes family demographics at time 1. There were no significant time 1 differences in family demographics or child T1D characteristics based on treatment arm. Likewise, there were no significant differences in time 1 parent FH or time 1 parent general anxiety. There was a significant group difference in time 3 parent FH. Specifically, parents randomized to REDCHiP reported lower time 3 FH than parents randomized to ATTN. At time 1, parents with elevated anxiety reported significantly higher FH (mean 64.1, SD 11.6) than parents without elevated anxiety (mean 53.3, SD 13.6; *P*<.001).

**Table 1.** Sample characteristics and outcome measures by treatment arm^a^.

	Overall (n=183)	REDCHiP^b^ (n=93)	ATTN^c^ (n=90)	*P* value
Demographics				
Child age at time 1 (y), mean (SD)	4.4 (1.2)	4.5 (1.2)	4.3 (1.2)	.12
Child sex, male, n (%)	100 (54.6)	51 (54.8)	49 (54.4)	.54
Parent age at time 1 (y), mean (SD)^d^	36.3 (5.9)	36.7 (5.3)	35.9 (6.6)	.26
Clinical characteristics				
Time since diagnosis at time 1 (y), mean (SD)^e^	1.7 (1.1)	1.5 (1.0)	1.8 (1.3)	.11
Continuous glucose monitoring use, n (%)	151 (82.5)	78 (83.9)	73 (81.1)	.36
Automated insulin delivery use, n (%)	93 (50.8)	45 (48.4)	48 (53.3)	.25
HFS-PYC^f^ total score at time 1, mean (SD)	56.4 (14.0)	55.4 (13.6)	57.4 (14.4)	.32
HFS-PYC total score at time 3, mean (SD)^g^	49.2 (11.0)	46.9 (9.5)	51.3 (12.1)	.009
Parent PROMIS-A^h^ total score at time 1, mean (SD)	54.6 (9.8)	54.8 (10.0)	54.4 (9.5)	.77
Parents with clinically elevated anxiety at time 1, n (%)	54 (29.5)	31 (33.3)	23 (25.6)	.17

a Full description of time 1 sample characteristics is reported elsewhere [12]; the variables reported here are relevant to the current secondary analyses.

b REDCHiP: Reducing Emotional Distress for Childhood Hypoglycemia in Parents.

c ATTN: attention control condition.

d Data were available for 177 participants overall (REDCHiP: n=90; ATTN: n=87).

e Data were available for 182 participants overall (REDCHiP: n=93; ATTN: n=89).

f HFS-PYC: Hypoglycemia Fear Survey–Parents of Young Children.

g Data were available for 152 participants overall (REDCHiP: n=77; ATTN: n=75).

h PROMIS-A: Patient-Reported Outcomes Measurement Information System Anxiety Short Form.

In our primary model, the treatment arm by anxiety interaction was not statistically significant (*F*_1,146_=2.40; *P*=.12; partial η^2^=0.016; Table 2). The main effect of REDCHiP was significant (*F*_1,146_=6.58; *P*=.01; partial η^2^=0.043), suggesting lower adjusted time 3 HFS-PYC scores among parents in the REDCHiP arm. Additionally, adjusted means showed larger absolute differences between REDCHiP and ATTN parents with clinically elevated anxiety (45.8, SD 1.75 vs 52.6, SD 2.12 for REDCHiP and ATTN, respectively) versus parents without elevated anxiety (48.6, SD 1.33 vs 50.3, SD 1.19 for REDCHiP and ATTN, respectively), though these subgroup patterns should be interpreted cautiously in light of the nonsignificant moderation effects.

**Table 2.** Analysis of covariance models examining time 1 anxiety and time 3 parent fear of hypoglycemia^a^.

Predictor	*F* test (*df*)	*P* value	Partial η^2^
Anxiety measured categorically			
Treatment arm	6.58 (1, 146)	.01	0.043
Elevated anxiety	0.013 (1, 146)	.91	0.001
Treatment arm×elevated anxiety	2.40 (1, 146)	.12	0.016
Time 1 HFS-PYC^b^	58.29 (1, 146)	<.001	0.285
Anxiety measured continuously			
Treatment arm	4.72 (1, 146)	.03	0.031
PROMIS-A^c^ (centered)	0.004 (1, 146)	.95	0.001
Treatment arm×PROMIS-A	1.48 (1, 146)	.22	0.010
Time 1 HFS-PYC	51.70 (1, 146)	<.001	0.261

a Both models included time 1 HFS-PYC as a covariate and 151 participants with complete data.

b HFS-PYC: Hypoglycemia Fear Survey–Parents of Young Children.

c PROMIS-A: Patient-Reported Outcomes Measurement Information System Anxiety Short Form.

In a follow-up model with time 1 parent anxiety measured as a continuous variable, the treatment arm by anxiety interaction was also not significant (*F*_1,146_=1.48; *P*=.22; partial η^2^=0.010). However, the main effect of REDCHiP remained significant (*F*_1,146_=4.72; *P*=.03; partial η^2^=0.031; Table 2).

Within the REDCHiP arm, 78.6% (22/28) of parents with clinically elevated time 1 anxiety met our responder criterion for reduced time 3 parent FH. In comparison, 47.4% (9/19) of parents with elevated time 1 anxiety in the ATTN arm met our responder criterion (Table 3). In an exploratory logistic regression among parents with clinically elevated time 1 anxiety and adjusting for time 1 FH, REDCHiP parents had greater odds of meeting responder criteria than ATTN parents (odds ratio [OR] 6.35, 95% CI 1.47-29.00; *P*=.01). For context, responders in both treatment groups reported higher time 1 FH than nonresponders (*P*<.001), suggesting that greater time 1 FH may have provided more opportunity for improvement. Among REDCHiP parents, responders also reported higher time 1 anxiety than nonresponders (*P*=.004).

**Table 3.** Fear of hypoglycemia responder rates at time 3 by treatment arm and time 1 parent anxiety.

Time 1 anxiety	REDCHiP^a^ parents		ATTN^b^ parents	
	Responders, n (%)	Nonresponders, n (%)^c^	Responders, n (%)	Nonresponders, n (%)
Elevated	22 (78.6)	6 (21.4)	9 (47.4)	10 (52.6)
Not elevated	17 (34.7)	32 (65.3)	28 (50)	28 (50)

a REDCHiP: Reducing Emotional Distress for Childhood Hypoglycemia in Parents.

b ATTN: attention control condition.

c Percentages are calculated within each treatment arm and anxiety subgroup. Subgroup sample sizes were n=28 (REDCHiP) and n=19 (ATTN) for elevated anxiety, and n=49 (REDCHiP) and n=56 (ATTN) for nonelevated anxiety.

## Discussion

In this secondary analysis of REDCHiP, we found that parents’ time 1 anxiety did not significantly moderate treatment response when modeled as either a categorical (*P*=.12) or continuous (*P*=.22) variable. However, exploratory results revealed a potentially meaningful pattern among parents with clinically elevated anxiety. Specifically, we found that adjusted time 3 FH scores were numerically lower in REDCHiP than in ATTN, and 78.6% (22/28) of REDCHiP parents with elevated anxiety met the responder criterion compared with 47.4% (9/19) of ATTN parents with elevated anxiety. Moreover, results of an exploratory logistic regression restricted to parents with elevated anxiety and adjusting for time 1 FH suggested that REDCHiP parents had 6-fold greater odds of meeting the responder criterion than ATTN parents.

We designed REDCHiP to address parents’ challenges with worry, hypervigilance, and avoidance related to childhood hypoglycemia [3,11]. While the current analyses did not support a significant moderating role for parent anxiety, the adjusted mean and responder findings suggest that it still may be worth considering parents’ time 1 anxiety symptoms when examining response to cognitive behavioral interventions for FH. One possible reason is that, although REDCHiP specifically targets FH, the cognitive and behavioral skills it teaches may generalize to broader, nondiabetes-specific worries and anxiety. As a result, it is possible that parents with elevated anxiety may derive benefits from REDCHiP that extend beyond FH. However, this interpretation remains speculative. We will need larger trials with adequate power to evaluate moderator effects to determine whether these observations reflect meaningful differences in treatment response.

The results also complement the primary trial outcomes, which showed that parents across both treatment arms experienced overall reductions in FH [12]. Whereas the primary trial focused on whether REDCHiP reduced FH, these secondary analyses examined variability in treatment response and provided additional context for interpreting response patterns among trial participants.

Strengths of this study include the use of validated questionnaires and sensitivity analyses examining anxiety as both a categorical and continuous variable. As a limitation, we acknowledge that any reported subgroup differences among parents with clinically elevated time 1 anxiety should be regarded as exploratory because neither treatment arm by anxiety interaction was statistically significant. We acknowledge a risk of bias because we used complete cases rather than an intention-to-treat approach. However, post hoc comparisons of trial completers versus noncompleters on time 1 demographic and clinical characteristics yielded no significant differences. Our responder analyses were also post hoc, exploratory, and for the logistic regression, were restricted to a small subgroup of our sample; they should be interpreted cautiously and considered hypothesis generating. Relatedly, we acknowledge that for both treatment arms, responders reported higher time 1 FH than nonresponders, suggesting that time 1 FH severity and greater opportunity for improvement may have contributed to responder classification and should be considered when interpreting the clinical significance of these findings. Although our sample reflects the demographic characteristics of families typically seen in pediatric T1D care centers in the United States [17], the findings may not generalize to families from other clinical settings. Replication in future samples powered specifically for moderation and recruiting families from more diverse clinical samples will be important.

In conclusion, time 1 parent anxiety did not significantly moderate treatment response. However, in exploratory analyses, we found that REDCHiP parents with clinically elevated anxiety demonstrated lower adjusted FH scores and higher responder rates than comparable ATTN parents. While these exploratory results should be interpreted cautiously, they point to a clinically interesting pattern that warrants investigation in future studies. Additionally, as digital and telehealth-delivered behavioral health interventions continue to expand within pediatric diabetes care, this study highlights a need for further research examining the patient or family member characteristics associated with treatment response and how to use this information to match families to interventions that may improve reach, efficiency, and impact.

## Supplementary material

10.2196/95475 Multimedia Appendix 1 Sample characteristics based on trial completers versus non-completers.

## Abbreviations

ATTN: attention control condition
FH: fear of hypoglycemia
HFS-PYC: Hypoglycemia Fear Survey–Parents of Young Children
IRB: institutional review board
OR: odds ratio
PROMIS-A: Patient-Reported Outcomes Measurement Information System Anxiety Short Form
REDCHiP: Reducing Emotional Distress for Childhood Hypoglycemia in Parents
T1D: type 1 diabetes
