# Supplementary material for: Parent Anxiety and Response to a Telehealth Intervention for Hypoglycemia Fear: Secondary Analysis of a Randomized Trial
Source: JMIR Pediatr Parent. 2026 Nov 3;9:e95475. doi: 10.2196/95475 (PMC13730211; doi:10.2196/95475)
Supplement: Supplementary file 1 [file pediatrics-v9-e95475-s001.docx]

| Supplemental Table 1. Time 1 Sample Characteristics Based on Completers versus Non-Completers | | | | | |
| --- | --- | --- | --- | --- | --- |
|  | Completers  (n=152) | Non-Completers  (n=31) | | p | |
| *Demographics* | | |  | |  |
| Child age, years, visit 1, mean ± SD | 4.4 ± 1.2 | 4.3 ± 1.4 | | .62 | |
| Child sex, male, n (%) | 83 (54) | 18 (58) | | .57 | |
| Parent age, years, visit 1, mean ± SD | 36.3 ± 5.9 *(n=149)* | 37.6 ± 7.3 *(n=30)* | | .46 | |
| *Clinical characteristics* | | |  | |  |
| Time since diagnosis, years, at visit 1, mean ± SD | 1.8 ± 1.1 | 1.5 ± 1.1 | | .28 | |
| CGM use, n (%) | 125 (82) | 28 (90) | | .47 | |
| AID use, n (%) | 80 (53) | 13 (42) | | .12 | |
| Parent Hypoglycemia Fear Survey total at Time 1, mean ± SD | 56.7 ± 13.3 | 54.8 ± 16.5 | | .54 | |
| Parent PROMIS-A total, mean ± SD | 54.6 ± 9.3 | 54.7 ± 11.6 | | .95 | |
| Parents with clinically elevated anxiety, n (%) | 47 (31) | 7 (22) | | .21 | |
